# Changes in Dietary Behavior, Physical Activity, and Mental Health During a Lifestyle-Based Wellness Program for Women Re-Entering the Community After Incarceration: A Single-Arm Pre-Post Program Evaluation

**DOI:** 10.1177/15598276261491723

**Published:** 2026-10-10

**Authors:** Sarah Rashdan, Sarah-Ann Keyes

**Affiliations:** 1 Albert Schweitzer Fellowship, Houston-Galveston Branch, Houston, TX, USA (SR); 2 3989 Department of Medicine, Baylor College of Medicine, Houston, TX, USA (SR, SAK)

**Keywords:** yoga, anxiety, meditation, exercise, nutrition, education, incarceration, reentry, health behavior change, self-efficacy

## Abstract

**Background:**

Women re-entering the community after incarceration experience substantial chronic disease and mental health burdens. Evidence regarding lifestyle medicine programming in transitional housing settings remains limited. This study evaluated changes in dietary behavior, physical activity, and mental health symptoms during a wellness program.

**Methods:**

This single-arm, uncontrolled pre-post program evaluation retrospectively analyzed existing survey data from 17 women residing at a transitional living facility in Houston, Texas. The program included weekly yoga and meditation sessions and monthly nutrition and cooking classes. Dietary behavior was assessed using an adapted 10-item Healthy Eating Assessment, mental health symptoms using the PHQ-4, and physical activity using frequency items from the IPAQ Short Form.

**Results:**

The adapted dietary assessment total increased from 28.9 to 39.7 points (mean change +10.8, 95% CI [8.4, 13.2]; *P* < .001). PHQ-4 total decreased by 0.9 points but was not statistically significant (95% CI [−1.9, 0.1]; *P* = .080). Secondary analyses showed improvements in several dietary items and all 3 physical activity frequency measures. In exploratory analyses, anxiety symptoms decreased whereas depression symptoms did not.

**Conclusions:**

Participation was associated with improvements in self-reported dietary behaviors and physical activity frequency. Larger controlled studies with validated measures and longer follow-up are needed.

“Perceived control, self-efficacy, and social support are possible mechanisms through which group-based programming could influence health behaviors.”

## Background

People with histories of incarceration experience a disproportionate burden of chronic and preventable conditions, including hypertension, asthma, arthritis, cervical cancer, and hepatitis.^1^ Longitudinal evidence also suggests that incarceration may be associated with worsening cardiovascular risk factors. A systematic review and meta-regression estimated mean increases of 5.3 kg in weight and 1.8 kg/m^2^ in BMI over 2 years, although findings for hypertension and other cardiovascular risk factors were inconclusive.^2^ Incarceration and community re-entry have also been associated with poorer self-reported health and adverse mental health outcomes, including depression and anxiety.^3,4^

Health literacy may further influence post-release outcomes. In a Transitions Clinic Network sample, approximately 60% of recently incarcerated adults had inadequate health literacy, which was associated with lower confidence in medication adherence and a greater likelihood of an emergency department visit before establishing primary care.^5^ Limited health literacy has also been associated with poorer health outcomes and greater healthcare utilization across adult populations.^6^ Together, physical inactivity, poor nutrition, and chronic psychological stress represent potentially modifiable pathways that align with lifestyle medicine domains, including physical activity, healthy eating, stress management, and social support.^7^

Community re-entry is a vulnerable period during which housing, employment, transportation, and other immediate needs may compete with chronic disease management and preventive care. Women re-entering the community after incarceration may encounter barriers involving continuity of care, insurance enrollment, prescription access, discrimination, and navigation of primary and preventive services.^8^ Recent criminal-legal system involvement has also been associated with increased hospital and emergency department utilization, including elevated hospitalization risk following release.^9,10^ Transitional living facilities may therefore provide a setting in which health-promoting behaviors and connections to care can be supported during re-entry.

Women involved in the criminal-legal system experience substantial mental health, substance-use, trauma-related, and other health needs that can complicate community re-entry.^11,12^ Programs developed primarily for men may not adequately address these needs, supporting gender-responsive approaches that incorporate individualized services addressing substance use, mental health, trauma, and broader social barriers.^12^ Evidence regarding health-promoting interventions for women during community re-entry nevertheless remains limited.

Angela House is a transitional living facility in Houston, Texas, serving women re-entering the community after incarceration. Residents progress through structured program levels and participate in core programming addressing topics such as women’s health, budgeting, and relationship recovery. A wellness program consisting of weekly yoga and meditation sessions and monthly nutrition education and cooking seminars was incorporated into routine programming. Surveys assessing dietary behavior, physical activity, and mental health were administered at baseline and 3 months for program-evaluation purposes.

Prior evidence supports further evaluation of these intervention components. A systematic review found that yoga was associated with reductions in anxiety symptoms, although study quality and heterogeneity limited certainty,^13^ and one study reported improvements in selected cardiovascular risk markers following a combined yoga and dietary intervention.^14^ Perceived control and opportunities for mastery may also influence health behavior engagement, particularly in populations experiencing constrained autonomy, although these constructs were not measured in the present evaluation.^15,16^

The contribution of this evaluation lies in its population and setting rather than in the novelty of the intervention components. We evaluated changes in self-reported dietary behavior, physical activity frequency, and mental health symptoms during participation in a structured wellness program among women residing in transitional housing after incarceration. We hypothesized improvement in the primary outcomes of overall self-reported dietary behavior and PHQ-4 total symptom burden. Individual dietary items and physical activity frequency were secondary outcomes, while anxiety and depression subscales were exploratory.

## Methods

This single-arm, uncontrolled pre-post program evaluation retrospectively analyzed existing survey data collected at prespecified baseline and 3-month time points during routine programming at Angela House. No research-specific data collection, participant contact, or intervention delivery occurred. The Baylor College of Medicine Institutional Review Board approved the study as minimal risk and granted waivers of informed consent and HIPAA authorization because the analysis involved existing program-evaluation records and no participant contact (protocol H-59040). This project was supported by the Albert Schweitzer Fellowship and Baylor College of Medicine.

Residents at Angela House progress through structured program levels, with level 1 representing the initial phase of residency. During level 1, residents were required to participate in core programming addressing topics such as women’s health, budgeting, relationship recovery, and other re-entry needs. The wellness intervention was incorporated into level 1 programming and included weekly yoga and meditation sessions of approximately 1 h and monthly hands-on nutrition education and cooking seminars. Concurrent residential services continued during the evaluation period and were not controlled or quantified.

Yoga sessions introduced accessible movement, breathing, mindfulness, balance, mobility, and strength-oriented exercises with adaptations for varying fitness levels. Nutrition sessions emphasized practical skill development through meal planning, food-label interpretation, balanced meal construction, affordable nutrient-dense foods, reduction of sugar-sweetened beverages and highly processed foods, and hands-on cooking demonstrations. Sample program content is summarized in Table 1.

**Table 1.** Sample Overview of Angela House Wellness Program Content.

Program Week	Yoga & Meditation Focus	Nutrition Education Topic^a^	Objectives
Week 1	Introduction to yoga, breathwork, Warrior I/II, Triangle, Extended Side Angle, Vinyasa foundations	​	Establish mindfulness, body awareness, breathing techniques, and foundational movement patterns
Week 2	Chair Pose, Low Lunge, Twist Variations, Half Split, Downward Dog, intention setting	​	Improve flexibility and mobility while introducing mindfulness practices
Week 3	Tree Pose, Dolphin Pose, Wide-Leg Forward Fold, Side Lunge, balance training	​	Develop balance, concentration, and mind-body awareness
Week 4	Side Plank, Grounded Side Plank, Pyramid, Horizon Lunge, Shiva Squat, advanced Vinyasa transitions	Building a Balanced Plate and Healthy Meal Planning	Introduce healthy meal construction alongside progressive strength and coordination training
Week 5	Back pain–relief sequence (Cat-Cow, Child's Pose, Pigeon, Twists, Forward Folds, Bridge)	​	Promote recovery, mobility, and stress reduction
Week 6	Goddess Pose, Locust Variations, Bridge, Side Plank, strength-focused flows	​	Increase muscular strength, stability, and self-efficacy
Week 7	Half Moon, Revolved Triangle, Gate Pose, Side Plank, balance progressions	​	Improve balance, stability, and confidence with more advanced movements
Week 8	Review and integration of prior sequences	Saturated Fat and Cardiovascular Health	Improve understanding of dietary fat quality and cardiovascular risk reduction

^a^Nutrition education sessions were delivered approximately monthly and incorporated hands-on cooking demonstrations and meal preparation activities.

Program design was informed in part by prior work evaluating the feasibility of physical activity and nutrition programming within community re-entry services.^17^ All residents were eligible to participate. Attendance at intervention sessions was required during level one but became optional after residents advanced. Participation in surveys was always voluntary, and declining participation did not affect housing status or access to services.

Eligible participants were Angela House residents aged 18 years or older who participated in the wellness program and completed at least one program-evaluation survey. Demographic and clinical characteristics beyond adult age eligibility and residence at Angela House were not systematically collected. Of 25 participants who completed baseline surveys, 17 completed the 3-month assessment and were included in paired analyses. The remaining 8 participants lacked follow-up data.

Three brief survey-based assessments were used to evaluate dietary behavior, physical activity frequency, and mental health symptoms. All assessments were administered at baseline and again at 3 months as part of routine program evaluation.

Dietary behavior was assessed using a 10-item Healthy Eating Assessment adapted from the validated eight-item Starting the Conversation screener.^18^ Items assessed overall eating habits and consumption of fried or fast foods, fruit, vegetables, sugar-sweetened beverages, snack foods, sweets, added fats, dairy products, and meat, fish, or beans. Each item was scored from 1 to 5, with higher scores representing behaviors designated as healthier, yielding a total score from 10 to 50. The adapted instrument and its composite scoring system have not been independently psychometrically evaluated in adults with histories of incarceration; scores were therefore interpreted as selected self-reported dietary behaviors rather than as a validated measure of comprehensive diet quality or clinical improvement. A prespecified sensitivity analysis recalculated the total score after excluding the dairy item because associations between dairy and cardiometabolic outcomes may depend on the product and comparator.^19,20^

Mental health symptoms were assessed using the validated four-item PHQ-4, comprising 2 anxiety and 2 depression items scored from 0 to 3.^21^ Higher scores indicate greater symptom severity. Anxiety and depression subscales were examined as exploratory outcomes, with scores ≥3 treated as positive screening thresholds rather than clinical diagnoses.^21^

Physical activity was assessed using items from the IPAQ Short Form, Last 7 Days Self-Administered Format.^22^ Analyses were limited to self-reported days per week of vigorous activity, moderate activity, and walking for at least 10 minutes. Complete paired frequency data were available for all 17 participants. Incomplete duration responses prevented calculation of standard IPAQ MET-minutes per week but did not reduce the sample size for the frequency outcomes. Participants included the program’s weekly yoga session in their responses.

Surveys were administered in paper format at baseline and 3 months. Attendance records were not systematically retained as part of the program evaluation and therefore individual intervention dose could not be quantified retrospectively. Responses were entered into a password-protected dataset; identifiers were retained only for linkage across time points and removed prior to analysis. All data were stored on secure institutional systems.

Outcomes were classified for analysis as primary, secondary, and exploratory. The primary outcomes were the adapted Healthy Eating Assessment total score and the PHQ-4 total score. A prespecified sensitivity analysis recalculated the adapted dietary assessment total after excluding the dairy item. Secondary outcomes included individual dietary-assessment items (sweetened beverages, vegetables, fruit, fried/fast food, and dairy) and physical activity measures from the IPAQ (vigorous, moderate, and walking days per week). Exploratory outcomes were the PHQ-4 anxiety and depression subscales. Because these analyses involved multiple comparisons and the study was not powered for subscale outcomes, the findings were considered hypothesis-generating.

Analyses were conducted in Python using SciPy and NumPy. Analyses were based on complete paired observations for each outcome, and outcome-specific sample sizes are reported in Table 2. Descriptive statistics were calculated at baseline and 3 months. The distribution of paired differences was evaluated using the Shapiro-Wilk test. Outcomes with approximately normally distributed paired differences were analyzed using paired t-tests and are summarized using means and standard deviations, mean paired changes with 95% confidence intervals, and paired-sample Cohen’s d_z_. Cohen’s d_z_ was calculated as the mean paired change divided by the standard deviation of the paired differences. Outcomes with non-normal paired differences were analyzed using Wilcoxon signed-rank tests and are summarized using medians and interquartile ranges, Hodges-Lehmann estimates of location shift with percentile bootstrap 95% confidence intervals, and matched-pairs rank-biserial correlations. Bootstrap confidence intervals were calculated by resampling participants with replacement at the paired-observation level using 500 000 replicates. Because Cohen’s d_z_ and rank-biserial correlations represent different effect-size metrics, they were interpreted within their respective statistical frameworks and were not directly compared. Statistical significance was defined as a two-sided *P* < .05.

**Table 2.** Full Results Summary.

Outcome	Baseline Mean (SD) or Median (IQR)	3-Month Mean (SD) or Median (IQR)	Change Estimate (95% CI)	Effect Size	*P* Value	Test
Primary outcomes						
Adapted healthy eating assessment total	28.9 (6.4)	39.7 (3.6)	+10.8 [8.4, 13.2]	*d*_*z*_ = 1.30	<.001	Paired t-test
Adapted healthy eating assessment total (excl. dairy)	26.6 (5.9)	37.2 (3.2)	+10.6 [8.3, 12.9]	*d*_*z*_ = 1.43	<.001	Paired t-test
PHQ-4 total	3.5 (2.0)	2.6 (1.3)	−0.9 [−1.9, 0.1]	*d*_*z*_ = 0.45	.080	Paired t-test
Individual dietary-assessment items (secondary outcomes)						
Sweetened beverages	3.0 (2.0, 4.0)	5.0 (4.0, 5.0)	+1.5 [1.0, 2.0]	*r*_*rb*_ = 1.00	.001	Wilcoxon
Vegetables	2.5 (1.3)	4.1 (0.7)	+1.5 [0.9, 2.2]	*d*_*z*_ = 0.94	<.001	Paired t-test
Fried/fast food	2.7 (0.9)	3.9 (0.8)	+1.3 [0.7, 1.9]	*d*_*z*_ = 0.99	.001	Paired t-test
Fruit	3.0 (2.0, 3.0)	3.0 (3.0, 4.0)	+0.5 [0.0, 1.0]	*r*_*rb*_ = 0.85	.013	Wilcoxon
Dairy	2.0 (2.0, 3.0)	3.0 (2.0, 3.0)	0.0 [−0.5, 1.0]	*r*_*rb*_ = 0.26	.360	Wilcoxon
PHQ-4 subscales (exploratory)						
Anxiety (items 1-2)	3.0 (2.0, 3.0)	2.0 (1.0, 2.0)	−0.5 [−1.5, 0.0]	*r*_*rb*_ = −0.71	.032	Wilcoxon
Depression (items 3-4)	1.0 (0.0, 2.0)	1.0 (1.0, 1.0)	0.0 [−0.5, 0.5]	*r*_*rb*_ = −0.09	.776	Wilcoxon
Physical activity (secondary outcomes)						
Vigorous PA days/wk (n = 17)	1.0 (0.0, 2.0)	2.0 (1.0, 3.0)	+1.0 [1.0, 1.5]	*r*_*rb​*_ = 1.00	<.001	Wilcoxon
Moderate PA days/wk (n = 17)	1.4 (1.3)	2.6 (1.6)	+1.2 [0.5, 2.0]	*d*_*z​*_ = 0.79	.004	Paired t-test
Walking days/wk (n = 17)	5.2 (2.0)	6.7 (0.6)	+1.5 [0.5, 2.4]	*d*_*z​*_ = 0.69	.006	Paired t-test

Normally distributed outcomes are presented as mean (SD), mean paired change with 95% CI, and paired-sample Cohen’s d_z_. Non-normally distributed outcomes are presented as median (IQR), Hodges-Lehmann location-shift estimate with percentile bootstrap 95% CI, and matched-pairs rank-biserial correlation (r_rb_). Bootstrap confidence intervals were calculated by resampling participants with replacement at the paired-observation level using 500 000 replicates. Positive dietary-assessment effect estimates indicate behaviors designated as healthier by the assessment. Negative PHQ-4 effect estimates indicate fewer symptoms at 3 months. Physical activity outcomes are reported as days per week and do not represent standard IPAQ MET-minutes. All outcomes included 17 paired observations. No adjustment was made for multiple comparisons.

## Results

Twenty-five participants completed baseline surveys, of whom 17 (68%) completed the 3-month assessment and were included in paired analyses; 8 participants (32%) lacked follow-up data. Follow-up was unavailable because participants either left the facility or advanced to a program level at which attendance was no longer required. Participant flow is shown in Figure 1. Baseline characteristics were unavailable for comparison between participants with and without follow-up data.

**Figure 1.**
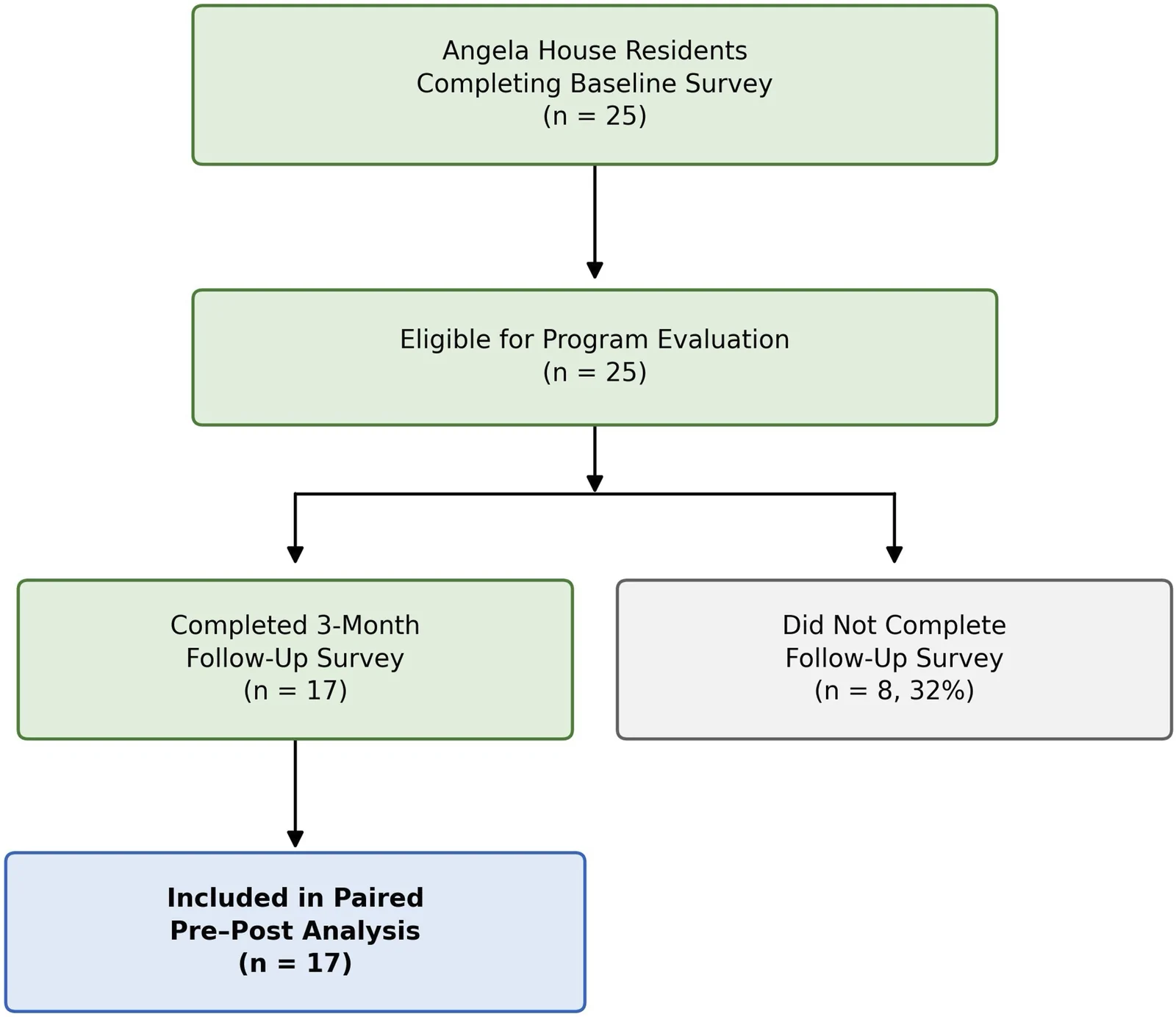
Participant flow diagram. Flow of participants through the wellness program evaluation. Twenty five participants completed baseline surveys, 17 completed the 3-month follow-up assessment and were included in paired analyses, and 8 did not complete follow-up.

### Dietary Behavior Outcomes

The adapted Healthy Eating Assessment total score increased from 28.9 at baseline to 39.7 at 3 months (mean change +10.8, 95% CI [8.4, 13.2]; *P* < .001; d_z_ = 1.30) (Figure 2). In the prespecified sensitivity analysis excluding the dairy item, the total score increased by 10.6 points (95% CI [8.3, 12.9]; *P* < .001; d_z_ = 1.43), indicating that the overall change was not dependent on the dairy item.

**Figure 2.**
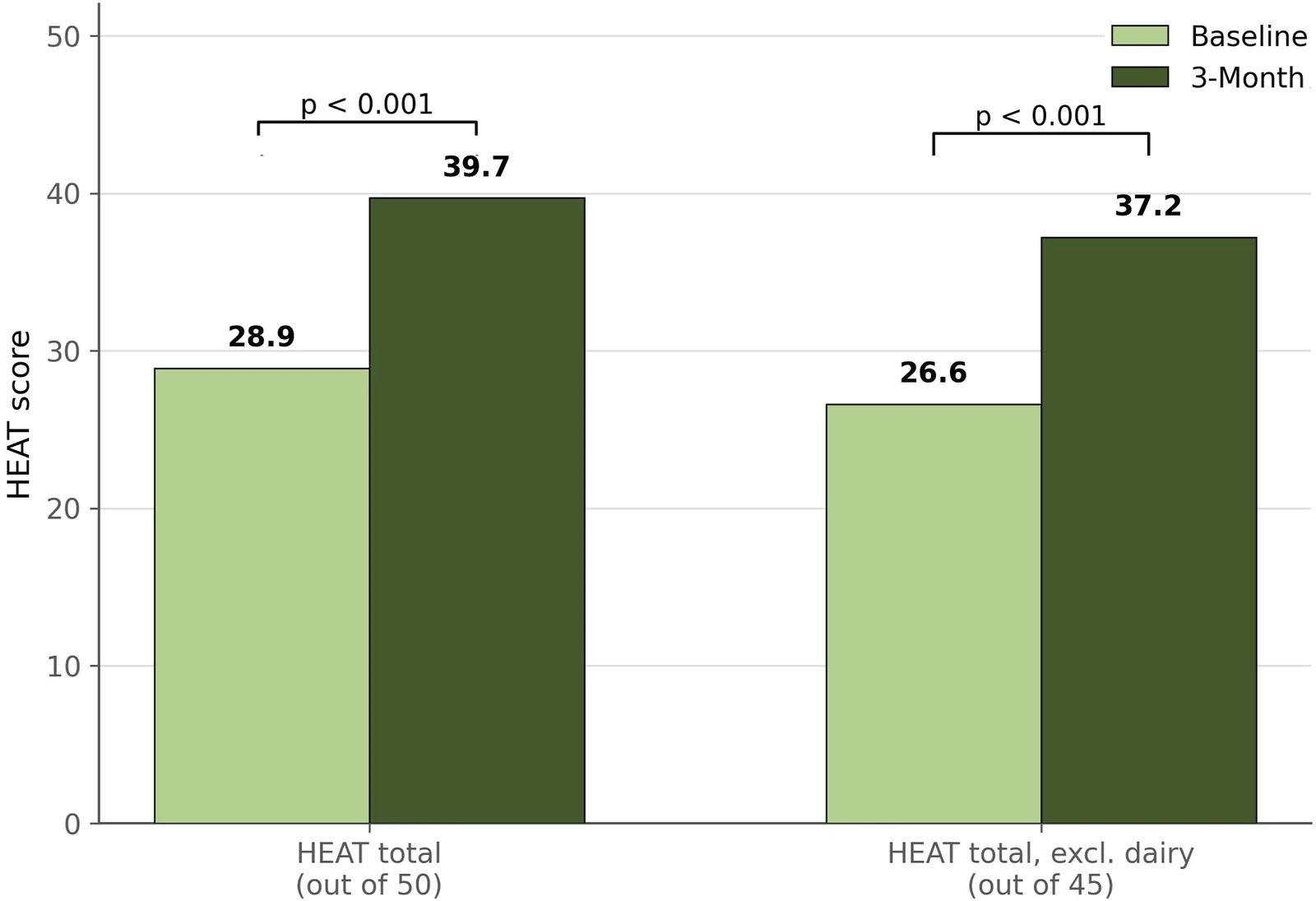
Adapted healthy eating assessment total scores at baseline and 3 months. Mean total scores for the adapted Healthy Eating Assessment, including the prespecified sensitivity analysis excluding the dairy item. Higher scores indicate dietary behaviors designated as healthier by the assessment. Error bars represent standard deviations.

Changes in individual dietary-assessment items are shown in Figure 3. The sweetened beverage score increased, indicating lower reported consumption, with a Hodges-Lehmann location-shift estimate of +1.5 points (bootstrap 95% CI [1.0, 2.0]; r_rb_ = 1.00; *P* = .001). Vegetable scores increased by a mean of 1.5 points (95% CI [0.9, 2.2]; d_z_ = 0.94; *P* < .001), and fried or fast food scores increased by a mean of 1.3 points (95% CI [0.7, 1.9]; d_z_ = 0.99; *P* = .001), with higher scores indicating more favorable reported behavior. The fruit score increased by a Hodges-Lehmann estimate of +0.5 points (bootstrap 95% CI [0.0, 1.0]; r_rb_ = 0.85; *P* = .013). The dairy score did not change significantly (Hodges-Lehmann estimate 0.0 points, bootstrap 95% CI [−0.5, 1.0]; r_rb_ = 0.26; *P* = .360).

**Figure 3.**
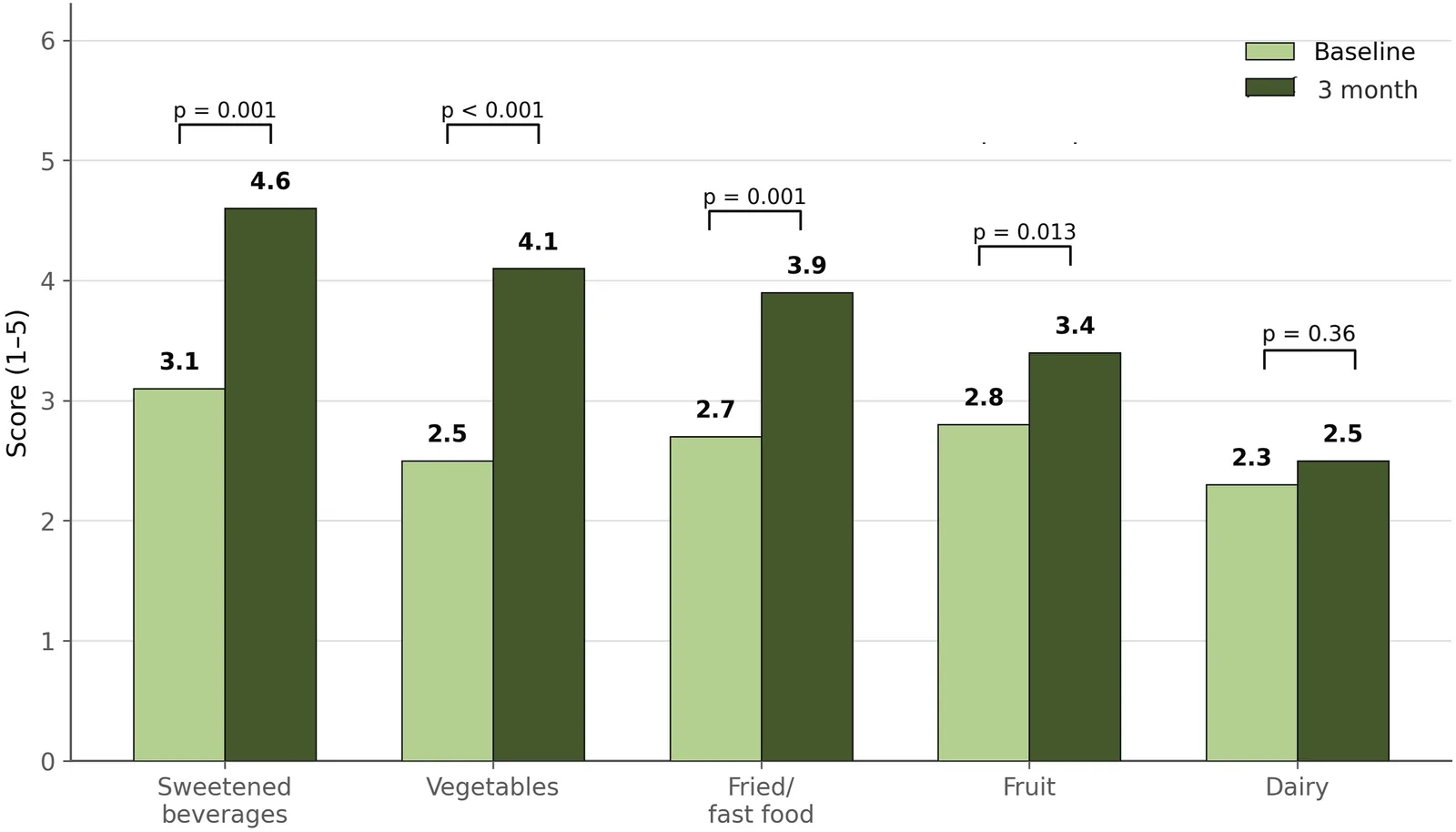
Individual dietary-assessment item scores at baseline and 3 months. Changes in selected dietary-assessment items from baseline to 3 months. Higher scores indicate dietary behaviors designated as healthier by the assessment. Values displayed are mean scores for visual comparison; statistical analyses are reported in Table 2. *P* values are shown for paired comparisons.

### Mental Health Outcomes

PHQ-4 total score was the primary mental health outcome, whereas the anxiety and depression subscales were exploratory outcomes. Among 17 participants with complete paired PHQ-4 data, the mean total score decreased from 3.5 at baseline to 2.6 at 3 months. This change was not statistically significant (mean change −0.9, 95% CI [−1.9, 0.1]; *P* = .080; d_z_ = 0.45) (Figure 4). In exploratory analyses, the anxiety subscale decreased by a Hodges-Lehmann estimate of −0.5 points (bootstrap 95% CI [−1.5, 0.0]; r_rb_ = −0.71; *P* = .032). Ten of 17 participants (59%) were at or above the anxiety screening threshold at baseline, compared with 3 of 17 participants (18%) at 3 months. The depression subscale did not change significantly (Hodges-Lehmann estimate 0.0 points, bootstrap 95% CI [−0.5, 0.5]; r_rb_ = −0.09; *P* = .776). Because the subscale analyses were exploratory and were not adjusted for multiple comparisons, these findings should be interpreted as hypothesis-generating.

**Figure 4.**
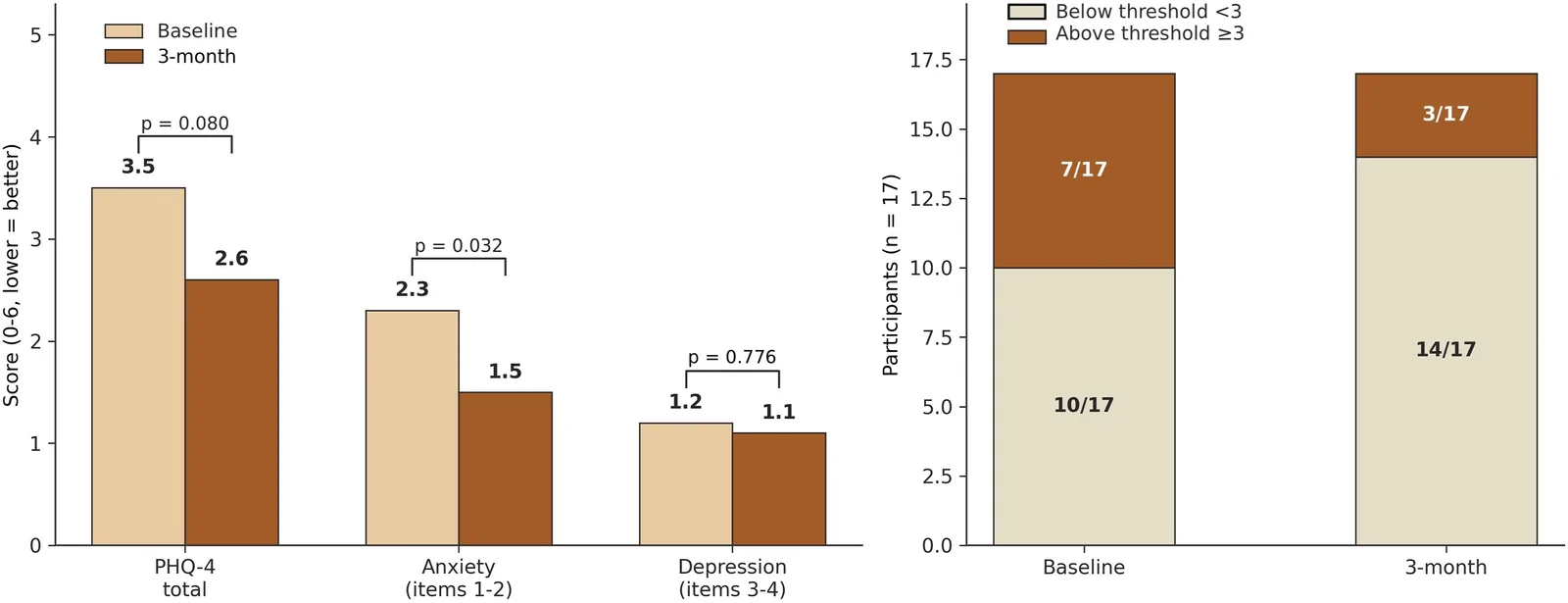
Mental health outcomes at baseline and 3 months. (A) Mean PHQ-4 total score and mean anxiety and depression subscale scores at baseline and 3 months. Lower scores indicate fewer symptoms. (B) Number of participants above and below the positive anxiety screening threshold (PHQ-4 anxiety subscale score ≥3) at baseline and 3 months. Anxiety and depression subscale analyses were exploratory

### Physical Activity Outcomes

All physical activity outcomes included 17 paired observations. Self-reported vigorous activity increased by a Hodges-Lehmann estimate of 1.0 day per week (bootstrap 95% CI [1.0, 1.5]; r_rb_ = 1.00; *P* < .001). Moderate activity increased by a mean of 1.2 days per week (95% CI [0.5, 2.0]; d_z_ = 0.79; *P* = .004), and walking increased by a mean of 1.5 days per week (95% CI [0.5, 2.4]; d_z_ = 0.69; *P* = .006) (Figure 5). These outcomes represent activity frequency rather than standard IPAQ MET-minutes per week.

**Figure 5.**
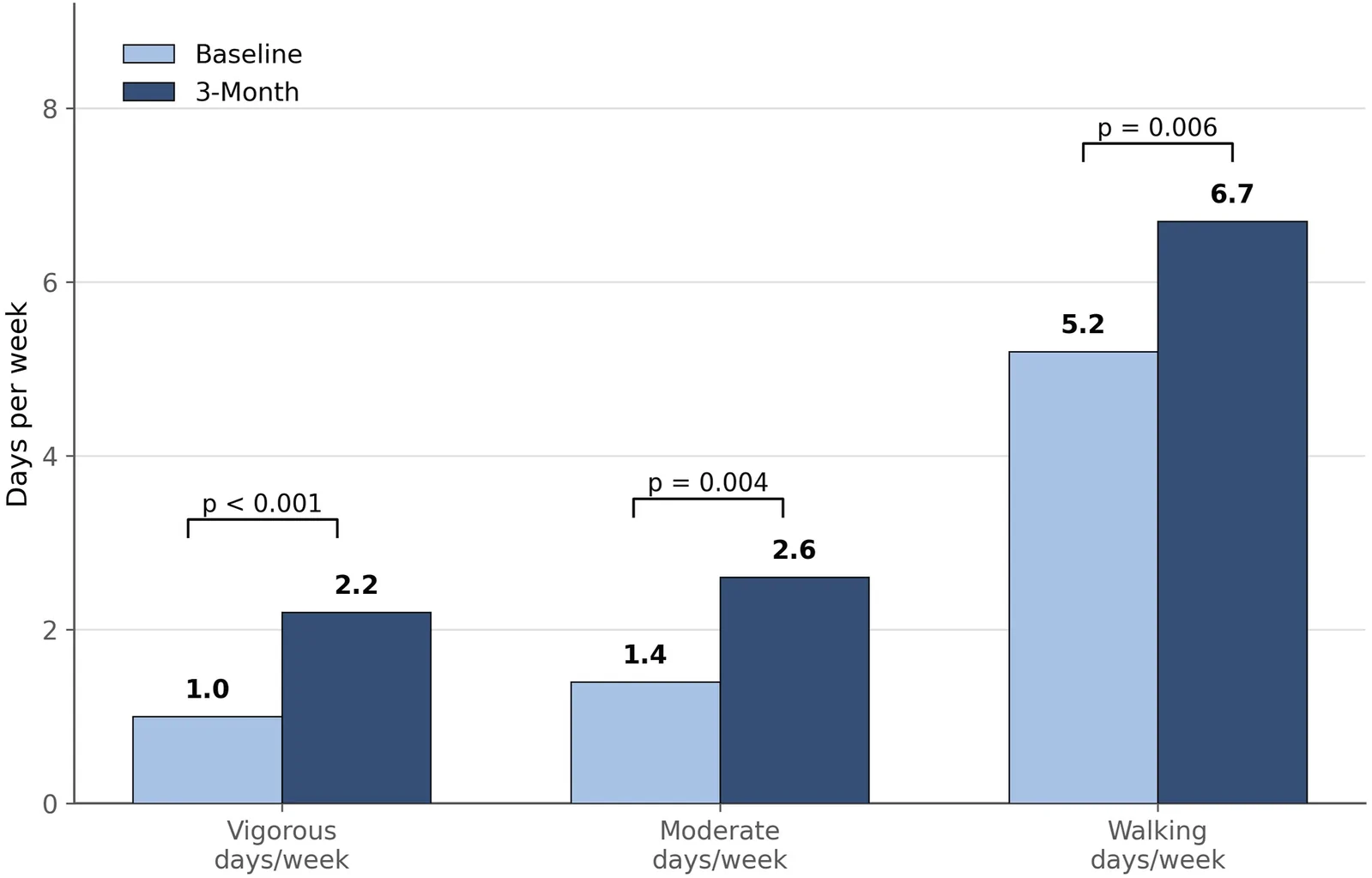
Physical activity frequency at baseline and 3 months. Self-reported vigorous physical activity, moderate physical activity, and walking frequency measured as days per week. Values represent activity frequency and do not reflect standard IPAQ MET-minutes per week. *P* values are shown for paired comparisons.

## Discussion

In this single-arm pre-post evaluation, the primary adapted dietary-assessment score and all 3 physical activity frequency measures improved over 3 months, whereas the primary PHQ-4 total outcome did not. Several individual dietary items improved, and an exploratory anxiety analysis yielded a statistically significant but unadjusted finding. Because the study lacked a control group, relied on self-report, and included only participants with complete follow-up, the observed changes cannot be attributed specifically to the wellness program.

The dietary findings were directionally consistent with prior cooking and combined lifestyle interventions.^14,23^ The program’s emphasis on hands-on food preparation, beverage substitution, and incorporation of vegetables reflected strategies used in other dietary behavior-change interventions.^24^ However, nutrition knowledge, cooking confidence, and other potential mediators were not measured.

Results were similar when the dairy item was excluded, suggesting that the composite-score change was not dependent on that item. Because evidence concerning dairy and cardiometabolic outcomes varies by product and comparator, and because the adapted dietary assessment has neither independent validation nor an established minimal clinically important difference, the total-score change should not be interpreted as a clinically meaningful improvement in comprehensive diet quality.^19,20^

The exploratory anxiety finding was unadjusted for multiple comparisons and should be interpreted cautiously. Its direction is consistent with literature examining yoga and exercise, including a randomized trial of yoga as an adjunctive intervention for posttraumatic stress disorder.^13,25,26^ The proportion meeting the positive anxiety screening threshold decreased from 59% to 18%, but the study cannot determine whether this reflected the wellness program, natural adjustment during re-entry, regression to the mean, concurrent services, changes in housing stability, or other unmeasured factors.

Depression scores did not change significantly. Low baseline scores may have limited the opportunity for reduction, although this interpretation is post hoc. The differing patterns in the anxiety and depression subscales may also have contributed to the smaller, non-significant PHQ-4 total change.

All 3 activity-frequency measures increased, but interpretation is constrained by the frequency-only scoring. The program itself provided a weekly yoga session that participants could count as activity, vigorous activity was low at baseline, and walking approached the 7-day upper bound at follow-up. The results therefore do not establish an increase in total activity volume and should be evaluated in future studies using complete IPAQ scoring or objective monitoring.

Perceived control, self-efficacy, and social support are possible mechanisms through which group-based programming could influence health behaviors. Meal preparation and accessible movement may provide mastery experiences, a theorized source of self-efficacy.^27^ Group participation may also provide opportunities for social connection, a factor associated with health outcomes more broadly.^28^ However, these constructs were not measured, and concurrent programming, natural adaptation during re-entry, regression to the mean, and response bias remain alternative explanations. Future studies should measure proposed mediators directly.

Program delivery and completion of paired surveys by 17 participants indicate that wellness programming and repeated outcome assessment can be implemented in this transitional housing setting. Whether the observed changes are sustained, generalize to other settings, or translate into objective clinical or healthcare-utilization outcomes remains unknown.

## Strengths and Limitations

This evaluation has several strengths. The paired design permitted estimation of within-participant change. The PHQ-4 and source IPAQ instrument have published validation evidence, and the program was evaluated in a real-world transitional housing setting. The generally consistent direction of observed changes supports further prospective evaluation.

The study lacked a concurrent comparison group, preventing attribution of changes to the wellness program rather than secular change, regression to the mean, concurrent services, or natural adaptation during re-entry. Individual attendance records were unavailable, preventing assessment of intervention exposure or dose-response relationships. Outcomes were self-reported, the sample was small, and secondary and exploratory analyses were not adjusted for multiple comparisons.

Eight participants (32%) lacked follow-up data. Complete-case analysis may have produced optimistic or otherwise nonrepresentative estimates if participants who remained available for follow-up were more engaged with the program, more stable in their re-entry process, or more likely to experience favorable outcomes. However, some participants became unavailable after advancing to program levels at which attendance was no longer required, and follow-up discontinuation did not necessarily indicate disengagement. Baseline demographic and clinical characteristics were unavailable, precluding assessment of differences between completers and non-completers and limiting evaluation of the direction and magnitude of attrition bias.

The single-site findings may not generalize to other settings. The adapted dietary assessment has not been independently validated and has no established minimal clinically important difference. Physical activity was measured as days per week rather than MET-minutes, and participants could count the program’s yoga session; the intervention may therefore account directly for part of the reported increase, and the measures do not establish change in total activity volume. No objective clinical or activity measures were collected, and the single 3-month follow-up precluded assessment of sustainability.

Future studies should use a prospective controlled design, a larger and more diverse sample, and longer follow-up. Priorities include validated dietary measures, complete IPAQ scoring or accelerometry, objective clinical outcomes, intervention attendance, and prespecified assessment of potential mediators such as self-efficacy, perceived control, and health literacy. Qualitative interviews could help identify valued program components and barriers to participation. If effectiveness is established, subsequent studies could evaluate implementation costs, healthcare utilization, and cost-effectiveness.

## Conclusion

Among 17 participants with complete follow-up, participation in a transitional housing wellness program was associated with improvements in selected self-reported dietary behaviors and physical activity frequency over 3 months. PHQ-4 total scores did not change significantly, and the exploratory anxiety finding should be considered hypothesis-generating. Larger controlled studies using validated dietary measures, objective or standard physical activity assessment, and longer follow-up are needed to evaluate effectiveness and sustainability.

## Data Availability

De-identified data are available from the corresponding author upon reasonable request, subject to IRB restrictions.*
